# Medical and Philosophical News

**Published:** 1782

**Authors:** 


### SECTION III.

### Medical and Philosophical News.

THE Royal Academy of Sciences at Paris have propoſed the following ſubject for a prize medal of 1080 livres value; viz. "To aſcertain "the nature and cauſes of the diſeaſes to which "gilders are expoſed, and the means of pre-

"venting them." The diſſertations are to be written either in Latin or French, and ſent to the Marquis de Condorcet, Secretary to the Academy, before the 15th of February, 1783.

The Academy having as yet received no ſatiſfactory account of the origin, ſtructure, courſe, termination, &c. of the lymphatics, originally propoſed as a ſubject for a prize, in the year 1779, have again announced it for the third time. The prize is a gold medal of 1500 livres value. It is expected of candidates that they ſhall deſcribe their experiments and apparatus with the greateſt accuracy. Diſſertations on this ſubject may be ſent to the ſecretary any time before November 1783.

---

At a public meeting of the Royal Academy of Surgery at Paris on the 11th of April, 1782, their prize medal of 500 livres value was adjudged to the learned Profeſſor Camper for his diſſertation on the effects of a morbid ſtate of the ſecretions in ſurgical diſeaſes, and the means of obviating them. This is the third time Dr. Camper has obtained the Academy's gold medal.

---

M. Poncelin,

M. Poncelin, ſurgeon at Aubreville near Clermont, has publiſhed an account of the ravages committed lately in that neighbourhood by a mad wolf. The animal was at length killed by a ſhepherd, who afterwards died mad in conſequence of the wounds he received. M. Poncelin found this poor man with the frontal and occipital muſcles in a great meaſure deſtroyed; the right eye-brow torn down over the eye; the lower lip carried away; the jaw bare; five teeth broken; the right cheek bit in two; a wound on the ſternum, which laid it bare; another two inches long in the fore arm, and reaching to the bone, and a third ſomewhat ſlighter in the right leg.

Mercurial frictions were employed for thirty days. The wounds healed, and on the 26th day he went home ſeemingly well. But about two days afterwards he was ſeized with a ſenſe of ſuffocation, which was ſucceeded by ſlight head-ach, convulſions, an averſion to food of every kind, whether ſolid or liquid, and likewiſe to light and any thing that was of a white color. The patient died within forty-four hours.—*Journal de Bouillon.*

---

Dr. Bergius, a celebrated Swedifh phyfician, has lately prefented to the Academy at Stockholm fome obfervations on the angina pectoris, and the method of treating it. He confiders the difeafe as a fpafmodic afthma, and contends that it deferves no other denomination. He has found a watery folution of gum guaiacum very fuccefsful in cafes of this fort. He triturates half an ounce of this medicine with two drachms of gum arabic, which he diffolves in nine ounces of diftilled water, adding to the whole half an ounce of fugar. Of this mixture he prefcribes one or two fpoonfuls to be taken night and morning, and the patient is directed to drink a pint of barley water after each dofe. This remedy produces daily one or two ftools, and it is by the number of thefe evacuations that the dofe is to be regulated.

---

One of the characteriftic properties of platina, hitherto, has been its infufibility by the moft intenfe heat. A little of it has indeed been melted by expofing it to the focus of a large burning glafs, but the moft violent heat which could be raifed in air furnaces, or by the united action of ſeveral large bellows, has never been ſufficient to bring it into a ſtate of fuſion. M. Lavoiſier, it ſeems, has ſurmounted this difficulty. One of our correſpondents at Paris, who was preſent lately at ſome experiments made by that celebrated philoſopher, on a new method of augmenting fire by means of dephlogiſticated air, informs us, that platina was very ſpeedily melted by this proceſs, and likewiſe that by the ſame means iron was made to detonate. Theſe experiments were made in the preſence of the Comte and Comteſſe du Nord at a meeting of the Academy of Sciences.

---

A great number of intereſting experiments on digeſtion have lately been made by the ingenious Abbé Spalanzani, Profeſſor of Natural Hiſtory at Pavia. He finds that a piece of fleſh incloſed in a glaſs tube, and ſwallowed by a crow, becomes putrid in nine hours, while another piece of the ſame ſize, that is ſuffered to be in contact with the coats of the ſtomach, remains perfectly ſweet for eighteen hours and upwards. From other experiments by the ſame profeſſor it would ſeem that the ſolvent power of the gaſtric juice does not laſt ſo long as its antiantiſeptic. Two glaſſes of this juice with a piece of fleſh in each were kept thirty-ſeven days in winter, without the leaſt mark either of putrefaction or diſſolution, whereas at the ſame ſeaſon ſimilar pieces of fleſh immerſed in water became putrid in ſeven days He finds that the gaſtric juice preſerves its antiſeptic power two months, and even then does not become putrid itſelf. He likewiſe proves that this juice corrects putrid fleſh, ſo that if an animal ſwallows putrid fleſh it becomes ſweet in the action of digeſtion.

His experiments agree with thoſe of M. de Reaumur with regard to the difficulty with which birds of prey digeſt vegetable ſubſtances. The eagle and vulture for inſtance eaſily digeſt fleſh, tendons, and even bones, but not corn or bread.

---

Mr. Kirwan, F. R. S. the learned and ingenious author of ſeveral chemical papers, is preparing a new table of elective attractions. This table will be conſtructed on a plan entirely different from thoſe which have hitherto appeared, and will afford ſingular advantages to the chemiſt. It will exhibit, for example, the ababsolute forces with which substances combine, and whether the combination will take place with or without heat. It will also include double elective attractions, the schemes for which are at present given separately, and are besides sufficiently puzzling to learners. By means only of common addition, or substraction, we shall be enabled, by this table, to know *a priori* what will result from a mixture of any of the chemical substances contained in it. To perfect this table a great number of experiments are now making by the author, in addition to those already made by others.

---

An ingenious physician in London, who has long taught midwifery with considerable reputation, intends soon to publish some observations to prove that neither the section of the symphysis pubis, or the Cæsarean operation, can ever be necessary. His ideas on this subject were suggested, it seems, by a case which occurred lately in his own practice, and at which several of the most eminent accoucheurs in London were present. In this case the diameter of the pelvis on one side, from the os pubis to the os sacrum, was sufficient to admit only a single finger; on the other fide, where its diameter was fomewhat larger, it was found not to exceed an inch and three quarters. As foon as this circumftance was fatisfactorily afcertained, and before the ftrength of the patient was at all impaired, the head of the fœtus was opened, and its brain evacuated. This operation, which was conducted with great care and deliberation, was completed in about two hours. The labour was then fuffered to go on, and on the fourth day, when the body of the child was in a putrid ftate, with its joints flexible, and the whole of it compreffible into a much fmaller compafs than before, it was brought away without any injury to the mother, who experienced no dangerous fymptoms after delivery, and was foon perfectly recovered.

---

A cafe of hydrophobia occurred lately at the General Difpenfary in Alderfgate-ftreet. The patient was a boy five years old, who was bit in the cheek two months before by a ftrange dog he was playing with in the ftreet. Nothing more was heard of the dog. When the phyfician, under whofe care the patient was admitted, firft faw him, he had an averfion to any thing liquid, liquid, ſwallowed it with difficulty, and ſeemed to be much affected by the cold air. He died the next morning.

---

On Tueſday the 30th of July his Grace the Duke of Montague, Preſident, attended by a great number of the Governors of St. Luke's Hoſpital for Lunatics, laid the firſt ſtone of a new building for that charity in Old-ſtreet Road. It is to be erected under the direction of Mr. Dance, on a plan nearly ſimilar to that of Bethlem Hoſpital.

---

Extract of a letter from Dr. Hahn, Profeſſor of Phyſic at Leyden, to Dr. Simmons, phyſician in London (dated Auguſt 18, 1782.)

" The ſpecies of bark you mention is em-

" ployed here under the name of *Cortex Peru-*

" *vianus Ruber*. It is of a deeper red colour,

" has more bitterneſs, and contains a greater

" proportion of reſin than the common bark;

" but I have not obſerved that it is ſuperior to

" that in efficacy. It was the ſecret of an apo-

" thecary at Amſterdam, who enriched himſelf

" by felling it in the form of a powder, to
" which he gave the name of *Antiquartan Spe-*
" *cific.* On examining this powder it might be
" immediately perceived that it confifted of bark,
" but its colour and bitternefs made people think
" there was fome other ingredient mixed with it.
" The art of the vender confifted in giving it
" in larger dofes than our phyficians ventured
" to prefcribe, or could eafily prevail on their
" patients to take. If you are in want of this
" fort of bark in England we can fupply you
" with it."

" Encouraged by the experiments that have
" been made in France, and particularly at
" Strafburgh, I have feveral times prefcribed
" the *Helminthocorton* or *Mufcus Corficanus*, and
" on my recommendation it has been repeatedly
" tried in this and the neighbouring towns. It
" is really the mildeft remedy I am acquainted
" with for common worms, and in general is
" very efficacious."

---

*Works in the prefs, or about to be publifhed.*——

1. A Latin edition of the late Mr. Hewfon's ingenious work on the Lymphatic Syftem, is now printing at Leyden. The tranflation is by Dr.

Dr. Thiens van der Wynperlfſe, ſon of the profeſſor of that name. The learned Profeſſor Hahn has undertaken to reviſe the tranſlation and prefix to it a preface, in which he means to give ſome account of the author. Materials for this purpoſe have been very obligingly furniſhed by Mr. Hewſon's family.——2. A volume, in 8vo, of intereſting eſſays on different ſubjects of midwifery, from the writings of M. M. Puzos, Levret, Camper, le Roi and Herbiniaux, is now printing in London, and will probably be publiſhed in the courſe of the preſent winter. Dr. Bland, who has undertaken to ſelect and tranſlate theſe pieces, means likewiſe to add notes to the whole. This collection will be very uſeful to accoucheurs, eſpecially to thoſe who are unacquainted with the French language.——3. A ſecond edition of Dr. Crawford's celebrated work on animal heat and combuſtion, is, we are aſſured, if not actually in the preſs, at leaſt in great forwardneſs, and will ſoon be publiſhed. The ingenious author has repeated his experiments with ſo much accuracy, that we have no doubt but every objection to his theory will be completely removed.——4. Dr. Alexander Monro's long-expected work on the ſtructure of the nerves is ſaid to be nearly ready for publication. Some

 account account of the difcoveries of this celebrated profeffor, relative to this fubject, may be feen in the fixth volume of Dr. Duncan's Medical Commentaries. Abbé Fontana, who, fince that account was publifhed, has directed his attention to the fame fubjects, agrees in many refpects with Dr. Monro, but differs from him in others. The reader will not be furprized at this, when he is told that thefe difcoveries are founded entirely on microfcopical obfervations.——5. A new edition (being the third) of the Medical Regifter is in the prefs, and will fpeedily be publifhed by J. Johnfon, bookfeller in St. Paul's Church-yard, London, where any corrections for that work will be thankfully received.

---

Several Phyficians and Surgeons, almoft all of whom belong to fome of the public charities in this Metropolis, have lately formed themfelves into *A Society for the improvement of Medical Knowledge.* They are defirous of collecting ufeful facts for publication. Several very interefting papers have already been read at their meetings, which are held once a fortnight, and it is hoped that practitioners in other places will be induced to contribute to fo ufeful a defign. Communications for this purpofe may be addreffed dreſſed to any of the following Gentlemen: Dr. Bromfield, Gerrard-ſtreet; Dr. Crawford, Lamb's Conduit-ſtreet; Dr. Douglas, Bedford-ſtreet, Bedford-ſquare; Mr. Ford, Golden-ſquare; Dr. Garthſhore, St. Martin's-lane; Dr. Gray, Britiſh Muſeum; Dr. Hicks, St. James's Palace; Mr. Howard, Southampton-ſtreet; Dr. Keir, Hatton-ſtreet; Dr. Oſborn, Percy-ſtreet, Rathbone-place; Dr. Simmons, Air-ſtreet, Piccadilly; Dr. Sims, Pater-noſter-row; Dr. Smyth, Charlotte-ſtreet, Bedford-ſquare; Mr. Watſon, Rathbone-place; Mr. Wyatt, Eſſex-ſtreet, Strand.

---

An account of the late Influenza is expected from the Royal College of Phyſicians in London. An addreſs from their Regiſter (Dr. Reynolds) to the Phyſicians of Great Britain and Ireland, has appeared in ſeveral of the public prints, requeſting them to communicate to him the reſults of their obſervations on this ſubject.—The Society inſtituted for the improvement of Medical Knowledge, a part of whoſe plan it is to compile an accurate regiſter of epidemical diſeaſes, are likewiſe collecting materials for a ſimilar hiſtory.

---

We remember to have heard Dr. Cullen, in one of his lectures, mention a remarkable circum-

ſtance

ſtance relative to the inhabitants of St. Kilda, one of the Weſtern Iſlands. This little body of people, which in 1764 conſiſted only of 88 perſons, have but little communication with the inhabitants of the neighbouring iſlands, and hardly ever ſee any other ſtranger than the ſteward belonging to Mr. Macleod, (the proprietor of the iſland) who once a year pays them a viſit to collect the rent, which is paid in ſheep and feathers. This viſitor never fails to prove a ſource of contagion to the inhabitants, who, within a few hours after his arrival, are univerſally ſeized with all the ſymptoms of contagious catarrh.—Dr. Macqueen, an ingenious phyſician at Yarmouth, who has had opportunities of making himſelf thoroughly acquainted with this curious fact, has lately communicated a circumſtantial account of it (in a letter relative to the late Influenza) to the Society inſtituted for the improvement of Medical Knowledge.—A fact of a ſimilar nature has occurred lately to the crews of the Convert and Lizard, two of his Majeſty's ſhips of war. Theſe frigates arrived in the river Thames, from the Weſt-Indies, in the beginning of September, upwards of two months after the late epidemical catarrh had entirely diſappeared in England. The crews of both ſhips were perfectly perfectly healthy till they reached Gravesend, where they took on board three custom-house officers, and in a very few hours after this the Influenza began to make its appearance. Hardly a man in either ship escaped it; and many, both of the officers and common seamen, had it in a severe degree.

